# Academic and social engagement outcomes of autistic students in general education classrooms: challenges with implementing inclusive practices to enhance quality of life

**DOI:** 10.3389/fpsyt.2026.1772732

**Published:** 2026-03-16

**Authors:** Karrie A. Shogren, Tyler A. Hicks, Hsiang Yu Chien, Lindsay F. Rentschler, Lauren Bruno, Kara A. Hume

**Affiliations:** 1 Kansas University Center on Disabilities, University of Kansas, Lawrence KS, United States; 2 Department of Applied Development Science and Special Education, University of North Carolina at Chapel Hill, Chapel Hill, NC, United States

**Keywords:** academic engagement, autistic adolescents, inclusive practices, peer support, self-determination, social engagement

## Abstract

**Introduction:**

Advancing inclusive supports that promote the academic and social engagement of autistic high school students is a priority. However, there is a need for comprehensive approaches that can address persistent implementation barriers. This study reports on the impact of a comprehensive intervention model that combined the Self-Determined Learning Model of Instruction and Peer Supports (SDLMI + PS). SDLMI + PS focuses on building supports for autistic high school students and their peers across general and special education.

**Methods:**

This study reports on the impact of a comprehensive intervention model that combined the Self-Determined Learning Model of Instruction and Peer Supports (SDLMI + PS). SDLMI + PS focuses on building supports for autistic high school students and their peers across general and special education. In this study, we examined the impact of this approach on autistic students’ academic and social engagement, as well as how able general and special educators were to implement this approach.

**Results:**

Overall, findings suggest that autistic high school students served primarily in general education classrooms experience low levels of social engagement and high levels of academic engagement throughout an academic year. The combined SDLMI + PS approach had small impacts on social and academic engagement worthy of future research when compared to SDLMI Only or PS only. Teachers, especially general educators, however, faced implementation barriers that warrant future attention to identify implementation strategies that can enhance the adoption and sustained use of collaborative practices that advance inclusion and quality of life as key priorities in high schools.

**Discussion:**

Future directions that respect the right to self-determination and social engagement, aligned with the strengths, visions, and priorities of autistic youth, are discussed.

## Introduction

Autistic high school students, as do all students, have a range of academic, social, and post-school priorities and goals (1). The supports that are provided during high school can be critical to enabling effective transitions to post-school environments, including postsecondary education, employment, and community living and participation, enhancing quality of life throughout adulthood. Researchers have established predictors of post-high school success for youth with disabilities (2). However, there is a need to enhance access to supports aligned with quality of life outcomes during high school, particularly for autistic students who are primarily served in general education classrooms, to enable more effective transitions. Providing effective supports requires a strong focus on bringing together general and special educators, autistic students, their peers without disabilities, families, and other members of school communities to advance inclusive supports aligned with enhancing quality of life (3, 4).

To address these priorities, the Propel Project, a large, federally funded research project implemented in schools across the United States, sought to bring together two evidence-based approaches for enhancing outcomes for autistic high students: Peer Supports (PS; 5) and the Self-Determined Learning Model of Instruction (SDLMI; 6). The goal was to combine these two evidence-based practices into a comprehensive intervention model (SDLMI + PS) that could be implemented across general and special education to advance inclusive supports leading to positive in-school and post-school outcomes, ultimately advancing inclusive practices and quality of life outcomes (Shogren, Hume, et al., in press^1^).

### SDLMI + PS

The SDLMI + PS comprehensive intervention model involves the SDLMI being delivered as designed by special educators during transition planning (6) while PS is delivered as designed by general educators during general education curriculum instruction (5). The only addition is SDLMI Plus, a brief version of the SDLMI, focused on group goal setting to be delivered class-wide by general educators to support all students to grow in their self-determination, linking what autistic students learn during more intensive special education supports with peer support arrangements and group goals in the general education classroom (Shogren, Hume, et al., in press).

#### SDLMI

Briefly, the SDLMI is designed to provide teachers with a model of instruction to engage their students in setting and going after goals for their learning, teaching students critical self-determination skills as they explore interests, identify and refine goals, build action plans, and recruit supports needed to navigate around barriers as they pursue goals throughout their high school education (6). Standardized SDLMI training and coaching enables teachers to engage students in 22 SDLMI lessons (approximately two 30–45-minute lessons per week) during an academic semester. SDLMI instruction is repeated across semesters, so students can refine their goals and action plans, building self-determination. SDLMI instruction is organized into three phases focused on supporting students to solve key problems in the goal setting and attainment process (Phase 1: What is My Goal? Phase 2: What is My Plan? Phase 3: What Have I Learned)?. SDLMI lessons and delivery are guided by three core components: Student Questions, Teacher Objectives, Educational Supports. There are four Student Questions per phase that guide students through the problem-solving process embedded in each phase. Teacher Objectives guide teachers in the steps they are taking in the lesson to support students to answer the Student Questions. SDLMI Educational Supports are built into each lesson and can be further intensified based on student need (e.g., providing supplemental instruction on skills like decision making, problem solving, self-advocacy that are targeted of the SDLMI Educational Supports if students need this to engage with the lesson content and Student Questions). The SDLMI has been widely researched, with demonstrated impacts on student in-school and post-school outcomes, including quality of life outcomes (7).pt?>

#### Peer Supports

PS focuses on building arrangements in general education classrooms to support students to work together to enhance academic and social supports (5). General educators are trained and coached to build a peer support plan with student input, identifying a peer group that sits in close proximity at least three times per week during the semester providing academic and social support to each other. An initial orientation meeting sets expectations with students for the peer support arrangements and autistic students and peers provide input that shapes the development of the PS plan and its implementation. Then data is collected by the teacher on the impacts. Efficacy of PS arrangements have been demonstrated with autistic students (8, 9), and there has been a growing focus on ensuring PS is reciprocal across students with and without disabilities (e.g., recognizing the value and contributions all students bring to peer supports, and that there are multiple ways to engage socially and support each other) leading to the inclusion of autistic students in identify peer groups and strategies used to implement PS plans. We specifically infused neurodiversity affirming practices into training and the creation of peer support plans in SDLMI + PS (Shogren, Hume, et al., in press).

#### SDLMI Plus

As noted, in addition to training and coaching special educators to deliver the SDLMI and general educators to deliver peer supports, the combined SDLMI + PS model also integrated SDLMI Plus, a set of six lessons focused on each phase of the SDLMI and group goal setting by the entire general education class for their learning. The six SDLMI Plus lessons can each be delivered in 15 minutes, and implementation schedules are created to align delivery across general and special education (e.g., Phase 1 lessons occur at the same time), supporting the transfer of content between instructional contexts and enabling autistic students to have multiple opportunities and supports to build self-determined goal setting skills.

### Purpose of this study

The Propel Project collected multiple sources of data from students, teachers, and schools over five years. One source of data was direct observations of autistic students conducted in general education classrooms to understand their experiences and to examine the impact of SDLMI + PS when compared to the other two conditions evaluated in the Propel Project: SDLMI Only (i.e., SDLMI delivered as designed by special educators with no general educator involvement) or PS Only (i.e., PS delivered as designed by general educators with no SDLMI Plus or special educator involvement). In this paper, we focus on social and academic engagement outcomes captured through observational data over a year of implementation of either SDLMI + PS, SDLMI Only, or PS Only. Given the importance of general and special educator engagement in SDLMI + PS to advance inclusive practices and supports aligned with student needs, we also explored teacher fidelity data from classroom observations. These observations were conducted by trained coaches three times each semester to better understand if and how general and special educators were able to initiate and implement SDLMI + PS. Other research from the Propel Project has noted significant implementation challenges that led to overall null findings on primary self-and teacher-reported outcomes (i.e. self-determination, goal attainment, and social contacts; Shogren et al., under review^2^); thus, further exploring if these null findings are also found in observational data and taking a more nuanced look at fidelity of implementation can inform ongoing international research to advance inclusive supports aligned with Article 24 of the Convention on the Rights of Persons with Disabilities (CRPD; 10), and integrate understandings of the supports model to enhance quality of life outcomes in inclusive schools (3).

Thus, we addressed the following research questions:

1. Are there impacts of the comprehensive intervention (SDLMI + PS) compared to SDLMI Only or PS only on social and academic engagement of autistic students in general education classrooms based on direct observation data from the classrooms?
2. How able were general and special educators to implement these interventions in their classrooms, based on fidelity observation data?

## Methods

### Setting and sample

The Propel Project took place in high schools in different regions of the United States (Midwest and Southeastern). High schools were recruited into the project in three cohorts. Each cohort implemented for one year (fall and spring semester) with supports from the research team (e.g., training and coaching). The first cohort entered the project in 2021-2022 (n = 12 schools) as students and teachers were returning to school after the COVID-19 public health emergency in the U.S. Cohort 2 (n = 11 schools) entered the project in 2022–2023 and Cohort 3 in 2023-2024 (n = 9 schools). After schools agreed to participate, they were randomly assigned to either the SDLMI + PS group, PS Only, or SDLMI Only. School-level random assignment was used as students were supported by multiple teachers and student or teacher random assignment was not feasible. As schools were recruited and randomly assigned on a rolling basis there were slightly imbalanced numbers across groups over the three cohorts (10 SDLMI Only schools, 10 PS Only schools, and 12 SDLMI + PS schools; 32 total). The schools varied in their locations and composition: 44% were in rural areas, 25% suburban, and 31% urban. Of the 32 schools that agreed to participate, 25 (81%) had available student observation data: 9 (90%) of 10 SDLMI Only schools, 9 (90%) of 10 PS Only schools, and 7 (58%) of 12 SDLMI + PS schools.

After schools agreed to participate, autistic students were identified that met inclusion criteria by their special education case managers (e.g., the staff that were responsible for supporting implementation of the student’s Individualized Education Program [IEP]). To be eligible, students had to have an IEP and a primary educational classification of autism. They also had to spend the majority of their day in inclusive general education classes with targeted special education support time that could focus on transition planning, consistent with the requirements of the Individuals with Disabilities Education Act. Between 1 and 8 autistic students were identified at each school (M = 4.43, SD = 2.64) for a total sample of 68 students, although only 59 (87%) had available classroom observation data that could be analyzed for this study. **Table 1** presents descriptive statistics for the 59 autistic students in the analytic sample. Students ranged in age from 14 to 17. The sample was predominantly male (85%), with smaller proportions identifying as female (10%) or non-binary (2%), and 3% with not reporting their gender identity. The majority identified as White/European American (59%), followed by Black/African American (17%), with smaller proportions identifying as Native American (3%) or multiracial (2%); 19% did not report their racial identity.

**Table 1:** Autistic student demographics by groups.

			Groups					
	Overall		PS Only		SDLMI Only		SDLMI + PS	
Characteristics	*N*	*(%)*	*N*	*(%)*	*N*	*(%)*	*N*	*(%)*
Total Analytic Sample	59	100%	19	100%	18	100%	22	100%
Gender Identity								
Female	6	10%	2	11%	3	17%	1	5%
Male	50	85%	14	74%	15	83%	21	95%
Non-binary	1	2%	1	5%	0	0%	0	0%
Unknown	2	3%	2	11%	0	0%	0	0%
Hispanic Identity								
No	47	80%	15	79%	15	83%	17	77%
Yes	5	8%	2	11%	0	–	3	14%
Unknown	7	12%	2	11%	3	17%	2	9%
Racial Identity								
Black/African American	10	17%	4	21%	3	17%	3	14%
Native American	2	3%	0	0%	1	5%	1	5%
White/European American	35	59%	13	72%	12	55%	10	53%
Multi-racial	1	2%	0	0%	1	5%	0	0%
Unknown	11	19%	2	11%	5	23%	4	21%
	M	(SD)	M	(SD)	M	(SD)	M	(SD)
Age	15.7	1.08	15.6	0.92	15.5	1.18	16.1	0.98

Percentages may sum to more than 100 due to rounding.

Teachers were recruited that were linked to the identified autistic students, based on the group to which the school was assigned. PS Only schools required general educators linked to the autistic student; SDLMI Only schools required special educators linked to the autistic student, and SDLMI + PS schools required a general and special educator linked to the same autistic student. In the SDLMI Only group 9 special educators participated; in the PS Only group 16 general educators; and in the SDLMI + PS group, 11 special educators and 14 general educators. Note in the SDLMI + PS group there were more general educators as some autistic students switched general education classes across the fall and spring semester and a new peer support plan had to be established. General and special educators were largely female (68% and 60%, respectively) and White/European American (100% and 85%, respectively). General educators had a range of training with 34% holding a bachelor’s degree, 28% a master’s degree, and 38% a master’s degree plus additional graduate coursework. Special educators included 20% with a bachelor’s degree, 40% a master’s degree, and 40% a master’s degree plus additional graduate training. General educators had, on average, 16.24 years of teaching experience (SD = 10.92) and reported 12.43 years of experience with autistic students (SD = 19.63), and special educators averaged 13.60 years (SD = 10.36) and reported 8.30 years of experience with autistic students (SD = 5.10).

### Research design

All study procedures were approved by university Institutional Review Boards (IRBs). As schools served as the unit of random assignment, the Propel Project implemented a cluster-randomized controlled trial (C-RCT; 11) to examine group (SDLMI + PS, PS Only, and SDLMI Only) effects. Consistent with Intent-to-Treat (ITT) principles (12), we used group assignment as our independent variable for Research Question 1, regardless of the status of implementation. This approach preserves the integrity of randomization and yields unbiased impact estimates (13). However, given the importance of implementation, we descriptively explore initiation, access to coaching, and fidelity of implementation in Research Question 2.

### Procedures

As described, schools and the students and teachers within them were randomly assigned to one of three intervention groups (SDLMI Only, PS Only, or SDLMI + PS). These interventions were briefly described in the Introduction. Specific procedures for the Propel Project for each group are described below. Across all groups, research team members served as trainers, coaches, and data collectors, supporting implementation as well as collecting direct observation data on student experiences in general education classrooms and fidelity data on teachers’ implementation through direct observation during coaching sessions.

#### SDLMI only

Special educators in schools assigned to the SDLMI Only group (n = 9) were trained to deliver the SDLMI during special education support time, consistent with established training protocols. Educators were expected to complete one SDLMI cycle (i.e., 22 lessons) during both the fall and spring semesters (two cycles total). Training was delivered at the beginning of the school year either in a 1½-day group format or a 4-hour individualized session, depending on recruitment timing and school needs. Trained research staff served as SDLMI coaches, following the established SDLMI Coaching Model (14). In this model, SDLMI coaches visit the classroom three times each semester, once during a lesson within each SDLMI Phase, to observe teacher instruction and provided feedback and action planning for ongoing implementation based on observational data. Coaches use the SDLMI Fidelity Measure (15) to structure their observation, rate fidelity, and provide structured feedback. The SDLMI Fidelity Measure includes items on diverse rating scales (yes/no, yes/partial/no, and Likert-type scales), capturing information on teacher adherence to the SDLMI core components, the quality of teacher delivery of the core components, and student responsiveness to the core components. Standards setting has been used to establish cut-scores reflecting adequate fidelity of implementation in each of these domains to impact student outcomes (16). The cut scores were used to evaluate the adequacy of teacher fidelity of implementation.

#### PS only

General educators in schools assigned to PS Only (n = 16) were trained to establish PS in general education classes either during a semester (if students switched classes each semester) or year (if classes were year-long). Four (22%) of the 18 autistic students in PS Only switched classes, necessitating a new general educator and peer support plan in the spring. Teachers received a standardized 1.5-hour training on PS, including (a) identifying and inviting peers for the peer support arrangement with input from the autistic student, (b) teaching the autistic student and peers to use strategies outlined in an individualized PS plan based on student needs in the classroom, and (c) supporting ongoing interaction and collaboration during class activities. Teachers were to facilitate PS arrangements at least three times a week for at least eight weeks during the semester. In this study PS arrangements were implemented in a range of inclusive classes, based on autistic student schedules, including core content classes (e.g., English Language Arts, mathematics) as well as other classes that high school students enrolled in as part of their program of study (e.g., nutrition, career and technical education, etc.). In each of these classes, consistent with established protocols (5) a small group of peers were identified by the teacher, with input from the autistic student, as having shared interests to establish the peer support group. Then a PS plan was developed by the teacher and students. Across classes, students that were part of the peer support arrangement sat in close proximity to each other and used strategies established in the PS Plan to support social (e.g., initiating conversations, making introductions to other classmates, conversing about activities) and academic (e.g., highlighting key concepts, sharing materials, providing feedback) goals during the class.

PS coaches observed implementation and provided feedback based on those observations three times per semester, completing the PS Fidelity Measure (17). The PS fidelity measure includes a range of items that measure adherence to core components and quality of PS arrangements with a target score of 80% for effective implementation.

#### SDLMI + PS

In the combined condition, the same training and coaching occurred as in the SDLMI Only or PS only group for special educators (n = 11) and general educators (n = 14), with the exception that general educators received an additional 1.5 hours of training on delivering SDLMI Plus and all training included content on building collaboration across general and special education. During training, an implementation schedule for the six SDLMI Plus lessons was created, aligned with the special educator’s SDLMI implementation schedule. As in PS Only, 5 (23%) of the 22 autistic student sample switched classes from fall to spring and had a new general educator in the spring necessitating new training and peer support arrangements. The same coaching and fidelity procedures were followed, with the addition of the SDLMI Plus Fidelity Measure during general educator observations, which added additional questions about implementation of the SDLMI Plus that were scored similarly to the SDLM Fidelity Measure, reflecting adherence to core components, quality of delivery, and student responsiveness.

### Direct observation outcomes

During the implementation year, additional, trained research staff visited a general education classroom of autistic students to collect direct observation data. Observers were not fully masked to the intervention conditions, but were not directly involved in implementation in the schools where they conducted observations. Teachers coordinated with the research team to schedule observations at representative instructional times during the day based on school schedules. The content of the classes where direct observations occurred varied based on autistic student’s schedules and included electives (22%), English Language Arts (13%), health (10%), history (19%), mathematics (19%), and science (17%). The study design called for three observation occasions during the school year (beginning of year, mid-year, end of year). Due to delays and implementation challenges, we collected lower than expected direct observation data, with 108 occasions from the 59 students (average 1.83 observations per student vs. 3 observations for students).

The Direct Observation Form (DOF) used in this study was embedded in an online data collection system to collect momentary time-sampling data. The DOF is an adaptation and extension of long-established procedures to collect momentary time sampling ecobehavioral data in classrooms capturing a range of student, teacher, and classroom characteristics impacting engagement (18), including adaptations to understand access to the general education curriculum (19) and the needs of students with complex support needs (20, 21). Trained DOF observers recorded autistic student behaviors every 30 seconds for 36 consecutive intervals. At the end of each 30 second interval, the observers coded the autistic student’s social and academic engagement across multiple dimensions (as well as other data). Academic and social engagement interval data were then used to develop indices of academic and social engagement for the observation that were used for analyses. As noted, this method has been extensively in research and provides reliable, ecologically valid data on students’ engagement in general education instructional contexts. To ensure reliability, a second observer was present for approximately 20% of all DOF sessions, with percent agreement exceeding 80%, calculated as the average interval percent agreement, indicating adequate reliability for research use.

#### Social engagement outcomes

To capture social engagement during the observation, two interval-level indicators were used. The indicators included whether the autistic student initiated social behavior toward another individual or received social behavior from another individual during the observation interval. These indicators were combined such that an interval was coded as socially engaged if either form of social behavior was present and coded as not engaged only when both were absent; intervals that were uncodeable (i.e., student could not be observed during a given interval for any reason) for one or both indicators were excluded. The resulting outcome represents a proportion score, ranging from 0 to 1, defined as the proportion of scored intervals in which the student was socially engaged during the observation.

#### Academic engagement outcomes

Academic engagement outcomes were also derived from interval-level codes, but the indicator focused on whether the autistic student consistently participating during the interval in the instructional activities of the class. Observers marked each interval as engaged, not engaged, or uncodeable. Academic engagement was also calculated as the proportion of scored intervals in which engagement was observed during the observation, ranging from 0 to 1.

Across social and engagement outcome data, missing interval-level data within observation sessions were minimal overall; proportions were calculated using only available intervals. Sensitivity analyses excluding students with >5% missing intervals within sessions yielded virtually identical results. Consequently, we retained all social and academic outcome data for this data analysis.

### Data analysis

Consistent with open science principles (22), analytic materials, data, and analytic scripts as well as other study information and materials, are available on OSF^3^.

#### Research question 1: social and academic engagement outcomes

The outcome data on social and academic engagement had a four-level hierarchical structure: 108 measurement occasions [level-1] nested within 59 students [level-2] (M = 1.83 observations per student, SD = 0.834), who were nested within 43 general and/or special educators aligned with their assigned condition [level-3] (3.50 students per teacher, SD = 1.871), who were within 25 schools [level-4] (4.43 teachers on average per school, SD = 2.637). This nested structure was managed using generalized linear mixed modeling (GLMM; 23). We separately regressed each engagement outcome on intervention group, using a three-part Beta GLMM since outcome measures yielded proportion data, to predict the growth of students over the academic year (approximately 10 months of intervention) across groups. Rather than simply using beginning, middle, and end of the year as categorical time points, we modeled time continuously using the month of data collection to better understand growth trajectories, because observations were collected at different times across schools and did not align to uniform measurement periods given implementation challenges. This specialized model utilizes a logit link for data falling inside the proportion interval (0, 1), with separate components accounting for outcomes exactly on either endpoint (0 and 1, respectively). Effects were expressed as Cohen’s h (the equivalent of Cohen’s d for proportion data), using h ≈ 0.30, 0.50, and 0.80 as benchmarks for small, moderate, and large effects. Standard distribution assumptions were applied for random effects at the student, teacher, and school levels. A benefit of multilevel modeling is its ability to manage different measurement occasions across students, thereby compensating for multilevel data dependencies while maximizing information under a Missing-At-Random (MAR) assumption (24).

Our primary analytic strategy for evaluating the impact of the intervention group on each type of engagement outcome using GLMMs was Bayesian Model Averaging (BMA; 25). BMA analysis provides a principled framework for comparing the posterior probability of competing models, overcoming limitations of traditional Null Hypothesis Significance Testing (NHST). We specified four candidate models by imposing different constraints on model effects: ( $M_{0}$) a null effect (i.e., no effects); ( $M_{1}$) a time-only effect; ( $M_{2}$) a group-only effect; and ( $M_{3}$) full effects (i.e., group, time, and interaction effects). While NHST typically aims to reject the null hypothesis ( $M_{0})$, BMA provides a calibrated interpretation of the evidence. Specifically, BMA assigns each model a relative probability, enabling probabilistic comparisons of rival models (26), Moreover, rather than basing predictions on a single model, BMA combines predictions across the set of theoretically motivated models, weighting by their relative support. This merging of predictions yields a more robust effect estimate, particularly useful when it is not feasible to select only one “best” model because of uncertainty resulting from low sample sizes or other factors.

We conducted BMA analysis in three steps. First, for each engagement outcome, we specified four competing models ( $M_{0}$, $M_{1}$, $M_{2}$ and $M_{3}$). Second, we evaluated the relative fit of the each model using *Deviance Information Criterion* (DIC; 27). In DIC analysis, the DIC value quantifies misfit. Third, these DIC values were converted into model weights using established BMA techniques (28). These weights represent the probability that this model affords the best balance of explanatory power and theoretical simplicity (29). In addition to model comparison, the probability of a group effect equals the sum of the weights of all models with the group effect (i.e., $W_{2}$ and $W_{3}$). For model evaluation purposes, model weights below 0.10 reflect minimal evidence; between 0.10 and 0.25, very weak but non-negligible evidence; between 0.25 and 0.50, weak evidence; between 0.50 to 0.75, moderate evidence; between 0.75 to 0.90, strong evidence; and above 0.90, very strong evidence (30). All models in this study were estimated with Bayesian estimation (with weakly-informed priors for computational stability) using a general-purpose Markov Chain Monte Carlo (MCMC) simulation procedure (31). MCMC simulation details, including priors and diagnostics, are available on OSF.

#### Research question 2: fidelity of implementation outcomes

To contextualize the findings from Research Question 1, which used ITT (i.e., analyses proceeded based on random assignment, not considering fidelity of implementation), we also explored available data for the teacher sample (n = 50) to understand how able general and special educators were to implement intervention components in their classrooms. We calculated and descriptively analyzed the expected and actual numbers of teacher implementers in the fall and spring, fidelity observations and coaching sessions in the fall and spring, and fidelity ratings across the SDLMI Only, PS Only, and SDLMI + PS groups.

## Results

### Research question 1: social and academic engagement outcomes

**Figure 1:**
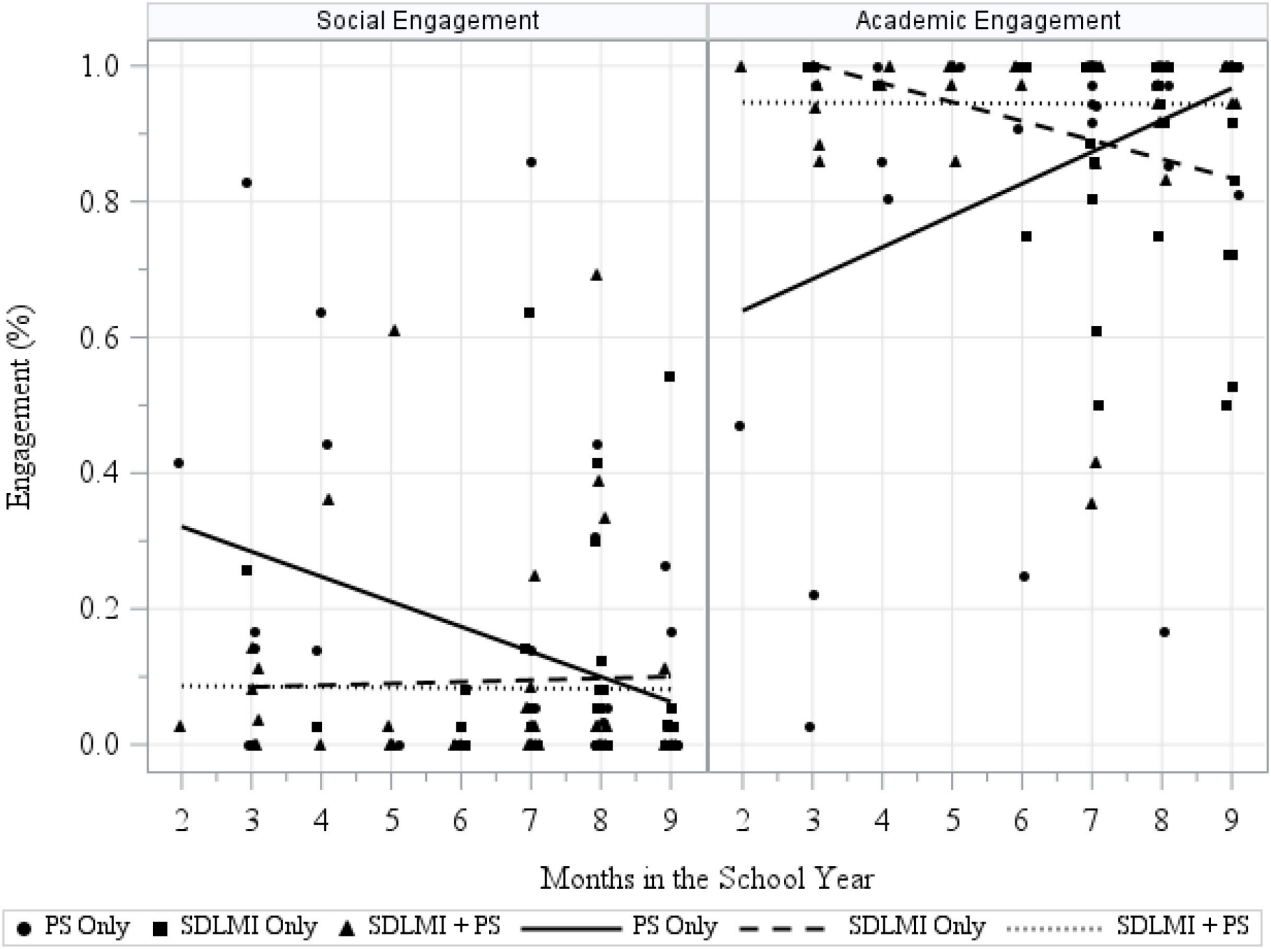
Visual plot of observed engagement outcomes. Observed social and academic engagement by group across the school year. Scatterplots depict individual engagement observations for students in the PS Only, SDLMI Only, and SDLMI + PS groups, with separate panels for social engagement (left) and academic engagement (right).

**Figure 1** illustrates longitudinal trends among observed data for both outcome measures, demonstrating overall low levels of social engagement across the year for autistic students across groups and relatively high levels of academic engagement across groups. **Table 2** provides an overview of BMA analysis based on these observed data across both social and academic engagement outcomes. Specific to social engagement outcomes, the left panel of **Figure 1** illustrates longitudinal trends in the observed data on social engagement across the three groups (PS Only, SDLMI Only, and SDLMI + PS). Descriptively, at baseline the PS Only group had the highest levels of social engagement with declines over time and the SDLMI Only and SDLMI + PS groups started lower and showed very slight gains. However, there was very sparce data in the early months (reflecting implementation initiation challenges, discussed subsequently) necessitating recognition of the potential impacts of low sample sizes on data patterns. When moving beyond the descriptive graph to compare different models, the time × group interaction model received the strongest weight ( $W_{3}=0.910$) although the group-only model ( $W_{2}=0.043$) and time-only model ( $W_{1}=0.042$) also had modest but similar support. The null model had negligible weight ( $W_{0}=0.005$). Although these model weights do not yet warrant selection of a single “best” model without more data, it is important to note that using BMA-predictions, the social engagement rates atter 10 months were 0.056 for PS Only group, 0.118 for SDLMI Only group, and 0.088 for SDLMI + PS group, suggesting higher rates of social engagement over time for the SDLMI and SDLMI + PS groups. Relative to the PS Only group, these group differences reflect a borderline small effect for SDLMI Only ( h=0.225) and a much smaller, yet non-trivial, effect for the combined group ( h=0.126), findings that warrant further investigation. However, it is critical to note that across all groups and across time, rates of social engagement started and remained very low for autistic students in general education classrooms.

**Table 2:** Model predictions derived from bayesian model averaging (BMA).

	$M_{0}$ empty		$M_{1}$ time only		$M_{2}$ group only		$M_{3}$ interaction		Effect sizes	
Group	Est	W	Est	W	Est	W	Est	W	E(R)	h
Social Engagement Effects										
PS	0.181	0.102	0.079	0.430	0.249	0.045	0.045	0.423	0.083	0.000
SDLMI	0.181	0.102	0.079	0.430	0.170	0.045	0.117	0.423	0.110	0.092
SDLMI + PS	0.181	0.102	0.079	0.430	0.132	0.045	0.086	0.423	0.095	0.042
Academic Engagement Effects										
PS	0.843	0.102	0.961	0.430	0.827	0.045	0.974	0.423	0.948	0.130
SDLMI	0.843	0.102	0.961	0.430	0.799	0.045	0.901	0.423	0.916	0.000
SDLMI + PS	0.843	0.102	0.961	0.430	0.909	0.045	0.988	0.423	0.958	0.175

E(R) denotes the expected rate after 10 months of intervention derived by pooling model predictions using BMA analysis; *h* denotes Cohen’s *h* (a standardized effect size for proportion data equivalent to Cohen’s d for metric data). Because $M_{0}$ and $M_{1}$ do not estimate any group effects, the first four columns are expected to be identical across groups.

In terms of academic engagement outcomes, the right panel of **Figure 1** illustrates longitudinal trends in observed data across the three groups (PS Only, SDLMI Only, and SDLMI + PS). At baseline, the PS Only group exhibited the lowest levels of academic engagement, but this level increased over time although, again, the small samples and sparce data must be considered in evaluating this data. In contrast, the SDLMI Only group began with higher baseline academic engagement but had slight decreases over time. By the final observations, academic engagement outcomes in both SDLMI-based conditions were lower than PS Only although all groups reflected high levels of academic engagement. Among the four candidate models, the time-only model ( $W_{1}=0.430$) and time × group interaction model ( $W_{3}=0.423$) received high and similar weights. The null model ( $W_{0}=0.102$) and the group-only model ( $W_{2}=0.045$) received considerably less support. Aggregating weights across models that include a given predictor yields strong evidence for a time effect ( $W_{1}+W_{3}=0.853$) and modest, but non-negligible, evidence for a group effect ( $W_{2}+W_{3}=0.468$). This pattern indicates that change over time may play a more prominent role than between-group differences in academic engagement. This is confirmed by the limited differences in BMA-predictions, as the academic engagement rates atter 10 months were 0.948 for PS Only group, 0.916 for SDLMI Only group, and 0.958 for SDLMI + PS group. Relative to the SDLMI Only group, these predicted rates suggest very small, yet non-dismissible, effects for PS Only ( h=0.13) and the combined group ( h=0.175), which warrant further investigation.

### Research question 2: fidelity of implementation outcomes

To contextualize these findings beyond the ITT analysis, we also explored implementation in the sample of teachers included in these analyses. **Table 3** provides an overview of teacher initiation of their assigned interventions across semesters, fidelity observations completed across the fall and spring semesters of the implementation year, and the percentage of teachers who met benchmarks for adequate fidelity of implementation over time.

**Table 3:** Fidelity observations and outcomes for PS Only, SDLMI only, and SDLMI + PS.

	PS only		SDLMI only		SDLMI + PS			
	General educators fall	General educators spring	Special educators fall	Special educators spring	General educators fall	General educators spring	Special educators fall	Special educators spring
Teachers Implementing	9 of 16 (56%)	16 of 16 (100%)	6 of 9 (67%)	9 of 9 (100%)	11 of 14 (79%)	14 of 14 (100%)	7 of 7 (100%)	9 of 9 (100%)
Number of Fidelity Sessions Conducted	12 of 27 (44%)	48 of 48 (100%)	16 of 18 (89%)	27 of 27 (100%)	21 of 33 (64%)	42 of 42 (100%)	19 of 21 (90%)	27 of 27 (100%)
Percent of Teachers Meeting Fidelity Standards								
PS Adherence*	72%	70%	--	--	59%	75%	--	--
PS Quality*	80%	74%	--	--	78%	84%	--	--
SDLMI Adherence*	--	--	100%	100%	--	--	100%	100%
SDLMI Quality**	--	--	88%	96%	--	--	84%	93%
SDLMI Responsiveness**	--	--	81%	100%			100%	96%
SDLMI Plus Adherence**	--	--	--	--	56%	56%	--	--
SDLMI Plus Quality**	--	--	--	--	81%	100%	--	--
SDLMI Plus Responsiveness**	--	--	--	--	81%	94%	--	--

In the PS Only group, in the Fall semester 9 of 16 general educators implemented PS, and in the spring all 16 trained general educators implemented PS. This reflects low initial adoption (56% of possible trained general education teacher implementers across the 25 schools). This also means that a large number of general educators did not receive coaching and feedback on fidelity of implementation in the fall, as they were not implementing. Further, students included in the analyses for Research Question 1 did not receive the full implementation of PS, as they did not receive both semesters of implementation. And even those general education teachers that did implement in the fall had fewer than expected (12 of an expected 27 [44%] fidelity observations and coaching sessions), reflecting delays in getting started in the fall semester, again limiting coaching and other supports to enhance implementation as well as student exposure to PS. All teachers, however, were able to implement and access coaching in the spring. And, when teachers did implement in the fall and spring, large majorities met the 80% standard for adherence to PS core components (72% fall and 70% spring) and quality of delivery (80% fall and 74% spring).

In the SDLMI Only group, proportionally more special education teachers began implementing in the fall (6 of 9; 67%). Further, there were close to expected numbers (16 of 18; 89%) of coaching and fidelity sessions in the fall with special education teachers, suggesting special education teachers may have been more able to initiate and follow implementation schedules than their general education peers. Further, when observed for fidelity sessions, special educators were largely able to implement with fidelity, with 100% of teachers meeting standards for adequate adherence to core components across fall and spring. Moreover, across fall and spring, a large majority of teachers met standards for quality of delivery (88% and 96%) and student responsiveness (81% and 100%), with increases from fall to spring, as expected with implementation experience.

In the SDLMI + PS group, relatively similar patterns were found across general and special educators. For both general and special educators, more began implementation in the spring than in the fall. Also, for general educators implementing PS and SDLMI Plus in the fall, there were lower than expected fidelity observations and coaching sessions (21 out of 33 expected; 64%), reflecting delays with initiating and being able to schedule fidelity and coaching. Fewer sessions were missed with special educators (19 out of 21 expected; 90%). In terms of actual fidelity outcomes, the fall in the SDLMI + PS group, general educators had much lower adherence for PS, but this increased in the spring, and quality of delivery remained relatively high. Fidelity for SDLMI Plus, delivered by general educators, however, was much lower, reflecting either a lack of implementation or a lack of full implementation of the core components (only 56% of teachers met standards for adherence to core components across fall and spring). However, when general educators did implement the SDLMI Plus core components, much larger numbers of general educators (81-100%) were able to meet standards for quality of delivery and student responsiveness. For special educators in SDLMI + PS, fidelity data were very similar to the SDLMI Only condition, with 84-100% of teachers consistently meeting standards for adherence, quality, and responsiveness across semesters.

## Discussion

This study examined social and academic engagement outcomes of autistic high school students in general education classrooms collected as part of the Propel Project, a large R-RCT that examined the impacts of an innovative SDLMI + PS model that sought to bring together general and special education supports to advance outcomes. In addition, we examined implementation data to contextualize findings and better understand general and special education teachers’ ability to implement evidence-based practices that are known predictors of meaningful outcomes in high school contexts. Our findings of relatively limited impacts of the SDLMI + PS model compared to SDLMI or PS Only and substantial variability in teacher implementation, highlight both the promise and the persistent challenge of advancing evidence-based inclusive practices to support quality of life outcomes for autistic high school students (32).

### Research question 1: social and academic engagement outcomes

Across intervention groups, autistic students demonstrated strikingly low levels of observed social engagement and strikingly high levels of observed academic engagement in general education classrooms throughout the academic year. Bayesian Model Averaging (BMA; 25) provided minimal support for differential trajectories across conditions. Specifically results suggested small increases in social engagement over time for groups that included the SDLMI relative to PS Only and smaller increases in academic engagement over time in groups that included PS relative to SDLMI Only. However, overall, levels of social engagement were minimal and academic engagement maximal throughout the year. In terms of social engagement, these findings align with prior research documenting limited social interaction for autistic students in inclusive high school classrooms (33). While small sample sizes and missing data must be considered in interpreting the results as outlined in the section on Limitations, our findings suggest directions for ongoing research. For example, the declines in the PS Only group may reflect common instructional practices and trajectories in high school classrooms, where the beginning of the semester often emphasizes relationship-building, classroom community norms, and cooperative learning structures. As the semester progresses, however, instructional priorities frequently shift toward content coverage and assessment preparation, especially in high school settings (34–36). High school schedules, curricular pressures, and accountability requirements may constrain the sustained development of social relationships, which is concerning given the well-documented importance of school-connectedness and peer relationships for adolescent well-being and longer-term quality of life outcomes.

While PS is meant to mitigate these barriers, our findings suggest there may be persistent implementation challenges that need to be addressed. For example, a greater focus on intentional and collaborative practices at the school-level, enabling reciprocal planning for and evaluation of peer supports as well as an inclusive school culture that fosters belonging are critical (32, 37). Further, in peer support arrangements and other interventions to support social engagement, there is a need for a greater focus on what autistic students bring and value and prioritize in their social interactions (38, 39). This is particularly important as research links social belonging and peer support to positive mental health, engagement, and post-school outcomes for autistic adolescents, while social isolation during adolescence is associated with poorer outcomes (40). Further as both the SDLMI and the SDLMI +PS group had slightly higher rates of social engagement over time, which was unexpected, ongoing research is needed to explore if there is some mechanism associated with fostering self-determination across general and special education that may equip autistic youth to engage in more social interactions aligned with their goal and priorities, above and beyond PS, particularly given research suggesting the importance of centering autistic youth’s priorities for their social interactions and quality of life (41).

In contrast, academic engagement was consistently high across groups. There were very small differences across groups and time. This pattern suggests that high school general education classrooms may be relatively successful at supporting autistic students’ engagement in academic activities which may be due in part to the often highly structured, standards-driven nature of high school coursework in the United States and many other countries (42, 43). High school classrooms are typically organized around clearly defined tasks, routines, and performance expectations which may align well with the learning strengths of many autistic students (44). High academic engagement can be associated with access to rigorous curricula, credit accumulation, and post-school pathways to higher education and employment (45); however, it is widely recognized that autistic young adults experience disparities in these outcomes, post-school (46). It may be that academic engagement without corresponding social engagement and broader inclusion risks reinforcing a form of “conditional inclusion, “ in which students are present and productive but not meaningfully connected to peers or school communities (47), negating the impacts of academic engagement on transition outcomes. Ongoing research is needed on the best constellation of supports to embed into comprehensive interventions like SDLMI + PS to build both academic and social engagement, as well as facilitate inclusive transition planning that brings together all members of school community to advance post-school outcomes (Shogren, Bruno, et al., in press^4^).

### Research question 2: fidelity of implementation outcomes

Findings suggest that there were implementation barriers that impacted teacher delivery of the PS, SDLMI Plus, and the SDLMI that likely impacted autistic students’ experiences and outcomes. Overall, as shown in **Table 3**; many teachers struggled to initiate implementation in the fall semester, with many postponing to the spring. This was most pronounced in PS Only group. However, even when teachers did initiate in the fall there were delays and other issues that often led to fewer than expected coaching visits and supports to enhance fidelity of implementation, which was again more pronounced for general educators. This is also reflected in the percent of teachers who were meeting standards for fidelity of implementation, which was notably lower for PS, particularly in the fall and for adherence to PS core components in the SDLMI + PS group. Further, general educators struggled with meeting adherence standards for SDLMI Plus, although they were able to demonstrate quality delivery and students were responsive when they did implement. This suggests adding another, new evidence-based practice to their classroom practice without broader support at the school-level was problematic. Overall, special educators seemed to be slightly more able to initiate in the fall, access coaching sessions, and meet standards for SDLMI adherence, quality, and responsiveness. This could be related to special educators being more familiar with models like the SDLMI, given its long history in special education and transition, as well as perhaps more flexibility in scheduling and special education and transition planning content.

These results point to the need for new and innovative implementation supports to bring together general and special educators to provide comprehensive and inclusive supports to benefit autistic students as well as their peers (32; Shogren, Bruno, et al., in press). Our findings highlight the difficulty of embedding complex, collaborative interventions in high schools (48). They also suggest that new and innovative implementation supports are needed to enable schools to use evidence-based practices that can impact student outcomes. For inclusive education to advance, in alignment with CRPD Article 24, interventions must not only be evidence-based but also implementable within the realities of high schools. This will require intentional structures that bring together general and special educators, administrators, student, families, and other school staff to share responsibility for inclusive practices (32). Without such coordination, comprehensive modules like SDLMI + PS may struggle to achieve their intended impact, necessitating a greater focus on innovative, inclusive models that advance quality of life and comprehensive supports in high school, and leverage what is known about effective implementation (32).

### Limitations and future directions

There are limitations that must be considered in interpreting the findings. First, the small sample size and missing data means the findings must be considered preliminary and a guide for ongoing research. We focused on probabilistic interpretations of the general directions of effect sizes rather than the exact effect size values, and replication in new and larger samples is needed. Second, implementation barriers led to considerable amounts of missing data at different levels of analysis. While there is something to learn from the implementation barriers, the missing data means that the final analytic sample may or may not be representative of the target population, and thus, caution must be exercised in generalizing findings. Third, as noted, ITT only looks at random assignment in interpreting outcomes. Given fidelity data, it is important to consider that many students were not exposed to the full assigned intervention and that the findings may reflect implementation challenges rather than intervention challenges. Fourth, our measures of social and academic engagement were limited to brief classroom observations and a relatively restricted number of indicators of engagement, which may not be representative of students’ experiences throughout their school day. Many meaningful peer interactions take place outside of academic instruction, such as during lunch, advisory periods, hallways, transportation, extracurricular clubs and activities, and unstructured time. Academic learning can also be shaped by other factors inside and outcome the classroom. Future research and practice should consider how to integrate a broader ecological perspective into definitions and data collection focused on academic and social engagement, to better understand how students themselves define and experience these outcomes to inform how social and academic engagement can be scaffolded across the school day and across school, home, and the community. Fifth, observers were not fully masked to study conditions and observations and peer support implementation occurred in a range of classrooms based on where autistic students were learning throughout the school day and which general educators were willing to support implementation and observations. Thus, future research needs to more fully explore teacher-level factors (e.g., experiences with supporting inclusive education and autistic students) and well as classroom factors (e.g., does the subject matter influence implementation and social and academic engagement)? Finally, for observation timing was coordinated with teachers to accommodate busy instructional schedules, which may introduce some selection bias in the context observed. Our results provide preliminary findings that can provide direction for future studies that should consider additional safeguards, such as fixed observation schedules or higher observation density, to further reduce potential contextual bias and better understand the experiences of autistic students, their peers, and the teachers that support them.

## Conclusion

This study aligns with research suggesting that autistic students in general education classrooms experience high academic and low social engagement, and that implementation barriers limit the ability of general and special educators to collaborate to advance comprehensive intervention models to address these issues (32, 37). Overall, these findings highlight that placement and academic classroom access alone are insufficient for meaningful inclusion, and that more thoughtful approaches are needed to build implementation supports for truly inclusive education and transition planning. This should include building collaborative and inclusive school cultures, centering autistic voices and the right to self-determination, and exploring how to structure supports to benefit all students through inclusive, school-wide models (Shogren, Bruno, et al., in press). Future efforts by researchers, practitioners, and policymakers should expand the focus to building school-wide contexts for social and academic engagement to overcome implementation challenges. To enhance quality of life and align with CRPD, we must work with the autistic community to better understand how to support and advance social engagement within high school communities. This should be done by collaborating with and considering the voices of all stakeholders to inform the school community, again aligned with autistic students, their peers, their families, and their school community’s self-determined visions and goals for the future.

## Data Availability

The datasets presented in this study can be found in online repositories. The names of the repository/repositories and accession number(s) can be found below: https://osf.io/qynph/overview?view_only=0ffe5c74eec14c33a6df20180550a65a.

## Footnotes

1 Shogren KA, Hume K, Rentschler L, Boyd B. (in press). Combining evidence-based practices to enhance outcomes for autistic students: Considerations and challenges. *Focus Autism Other Dev Dis*. doi: 10.1177/10883576251407633

2 Shogren KA, Hume KA, Hicks TA, Rentschler LF, Chien HY, Alsaeed A, et al. (2025). Examining the impacts of integrating peer support and self-determination interventions for secondary autistic students: An intent-to-treat analysis. Manuscript under review.

3 https://osf.io/qynph/overview?view_only=0ffe5c74eec14c33a6df20180550a65a.

4 Shogren KA, Bruno L, Taconet, A, Scott L, Mazzotti VL. (in press). Advancing inclusive transition planning: challenging our models. *Int J Dis Dev Educ*. doi: 10.1080/1034912X.2025.2528188
